# A Hydro-Mechanical Theory of Sensitive Dentin

**Published:** 1900-01-15

**Authors:** G. S. Junkerman

**Affiliations:** Cincinnati


### A HYDRO-MECHANICAL THEORY OF SENSITIVE DENTIN.

BY G. S. JUNKERMAN, D.D.S., CINCINNATI.

Read before the Ohio State Dental Society, December, 1899.

It is not the object of this paper to travel any new and undiscovered country, but rather to turn backward on the track of discovery and glean from the wreck of discarded fads a few ideas for the intelligent application of dental therapy. It is a false premise to assume that a practical man is independent of theory. A theory usually precedes a good practice. A perfect balance between the two is requisite always to a trusty practitioner. Theories may exist that can not be proven, much less disproven. No theory, however, is tenable that does not satisfy all the phenomena connected with it.

Cataphoresis had its fling, being based on the supposition that it was a local anesthetic. Having failed in this department of therapy it was eminently successful in another, giving a most excellent account of itself as a pulp destroyer. Cataphoresis may not have brought to light the fountain of eternal painless dentistry, but its intimate study has caused a new light to fall and while it may not have been the goal, it certainly has been a path-finder. Cataphoresis carries with it the thought of an osmosis, the track of dentin being an osmotic membrane, the pulp on one side, and the external fluids on the other. We think of the dental tubuli, but we forget the nerve fibrillæ. The accepted truths of cataphoresis prove the dental tubuli to be filled with fluid and not fibrillæ. The next step is to account for the phenomena of sensitive dentin, conceding the absence of fibrilæ. Impressions must be conveyed to the pulp which is the seat of all sensation in the teeth by means of hydro-mechanics; pressure, or motion of fluids in tubes. Picturing to yourself the principle of the hydrostatic paradox and using the pulp-chamber with its contents as the vessel, and one of the dental tubuli as the tube we have a reproduction of the hydrostatic paradox. Pressure produced on the end of the tube will be equally distributed to the contents of the pulp-chamber, whether the pressure be produced by cotton or the stroke of an instrument. If you put a bundle of these together you have an osmotic membrane upon which impressions outward or inward may be made by any substance that will induce osmosis. Sugar and salt produce pain upon the pulp through these tubes by disturbing the equilibrium of the fluid in them, producing traction or compression. The condition known as teeth being on edge is another exemplification of the same principle. The continued use of acid when the teeth are "on edge" is not productive of pain, but the instant an alkaline food is brought in direct contact with the teeth the osmotic influence is changed, and a disturbance in the tubuli occurs with resulting pain. In furtherance of the same phenomenon, if a hard body, as the smooth surface of a steel instrument, be brought in contact with the "on edge" surface no pain

(*To be Continued.*)

